# CDC42EP3 promotes glioma progression via regulation of CCND1

**DOI:** 10.1038/s41419-022-04733-9

**Published:** 2022-04-01

**Authors:** Zhigang Yang, Tao Xu, Tao Xie, Liangliang Yang, Guiping Wang, Yang Gao, Gangming Xi, Xiaobiao Zhang

**Affiliations:** 1 Department of Neurosurgery, Zhongshan Hospital, Fudan University, Shanghai, China; 2 Department of Neurosurgery, Changzheng Hospital, Naval Medical University, Shanghai, China; 3 Department of Neurology, Shanghai Xuhui Central Hospital, Shanghai, China

**Keywords:** CNS cancer, Molecular biology

## Abstract

Gliomas are the most common brain malignancies characterized by high degree of aggressiveness and high mortality. However, the underlying mechanism of glioma progression remains unclear. Here, we probed the role of CDC42EP3 (CDC42 effector protein 3) played in glioma development and its potential downstream mechanism. The expression of CDC42EP3 in tumor and normal brain tissues were examined through immunohistochemistry and we found the likelihood of CDC42EP3 overexpression was positively correlated with pathological grading. Patients with higher expression of CDC42EP3 were more likely to suffer from recurrence as well. Through constructing CDC42EP3-knockdown cell models, we discovered that silencing CDC42EP3 significantly restricted cell proliferation and migration but facilitated cell apoptosis in vitro. Inhibition on tumor growth mediated by CDC42EP3 depletion was further verified in vivo. Regarding downstream target of CDC42EP3, we found that it may positively regulate the expression of CCND1 through c-Myc-mediated transcription. Furthermore, our findings affirmed that effects of CDC42EP3 overexpression on cell proliferation, migration and apoptosis could be confined by depleting CCND1. In a word, this study reported the tumor-promoting role of CDC42EP3 in glioma progression which probably functioned through targeting CCND1.

## Introduction

Gliomas occupy almost 80% of primary brain malignancies and are supposed to be the most common intraparenchymal tumors derived from the central nervous system [1]. In accordance to classification defined by World Health Organization (WHO) in 2016, gliomas are individually into low grade gliomas and high-grade gliomas based on both histological and cytological characteristics [1]. Moreover, the new version of WHO classification of gliomas establishes some different approaches to both central nervous system (CNS) tumor nomenclature and grading with the introduction of a lot more tumor types and subtypes [2]. Generally speaking, the high-grade gliomas are generally characterized by high incidence rate and death rate [3]. In addition, patients with gliomas may also suffer from neurocognitive impairment which was suggested to connect with glioma location [4]. Through a systematic meta-analysis, scholars found that frontal gliomas were significantly relevant to a high morbidity of preoperative seizures [5]. Up to now, molecular characterization and their biologic significance have increased and restructured our understanding of gliomagenesis and tumor progression [6–8]. Despite recent substantial improvements in diagnostics and progress in aggressive therapy, median survival of patients with glioblastomas (grade IV) can be lowest as 8 months (14~16 months) following treatments [9, 10]. Lack of efficacy of therapy urges the need for the exploration of underlying mechanism and cancer-promoting genes in glioma progression.

The small GTPase CDC42 (cell division cycle 42) which belongs to the Rho family regulates diverse signaling pathways and participates in multiple biological procedures, for example cell migration, cell motility, cell cycle progression and cell endocytosis. A recent study reviewed that hyperactivation of CDC42 signaling was suggested to be involved in oncogenic processes [11, 12]. CDC42EP3 (CDC42 effector protein 3) is one of five CDC42 effector proteins and functions as a key regulator of the activities of CDC42 [13]. It has been proved that DNA damage response signaling could be triggered by overactivated CDC42 via CDC42EP3/Borg2 protein partners under circumstance of genome instability [14]. The correlation between increased CDC42EP3 and tumor progression has been identified in several tumor types. A recent study on gastric cancer reported that CDC42EP3 was upregulated in tumor tissues and high level of CDC42EP3 was also connected with the tumor pathological grade. Following CDC42EP3 knockdown, cell growth and migration were significantly repressed while the apoptotic cells were considerably increased due to upregulation of pro-apoptotic proteins [15]. Similarly, upregulated CDC42EP3 was identified in colorectal cancer cell lines (i.e., HCT116 and RKO) and inhibition in tumor cell growth and migration in vitro could be attributed to CDC42EP3 knockdown. Restrictions on in vivo tumor growth were also testified following depletion of CDC42EP3 [16]. However, the role played by CDC42EP3 in human glioma development is not yet clear.

Herein, we examined the expression of CDC42EP3 in human glioma clinical samples and in vitro alterations in cell proliferation, cell cycle, cell migration and apoptosis after building CDC42EP3-knockdown cell models via lentiviral transfection. Mice models were also established to verify the effects of CDC42EP3 depletion on tumor growth. In addition, downstream molecular mechanisms related to CDC42EP3 in cell models were also investigated. Our findings provided preliminary insights into regulation of CDC42EP3 in human glioma and available references for the development of targeted therapy in glioma.

## Materials and methods

### Clinical sample analysis

Tumor tissues (*n* = 165) from patients with glioma and normal brain tissues (*n* = 24) were included in the tissue microarray for immunohistochemistry analysis (IHC). Specifically, the formalin fixed and paraffin embedded tissues microarray were baked in oven at 65 °C for 30 min. After deparaffinized and rehydrated, the sections were digested by citric acid solution for antigen repairing and blocked with 3% H_2_O_2_ for endogenous peroxidase inactivating. Target antibody (CDC42EP3, 1:50, Cat. #NBP1-88382, NOVUS) and the corresponding second biotinylated antibody was added. DAB method and Hematoxylin were applied for staining and IHC scores were determined depend on the staining percentage and staining intensity. Specifically, the staining percentage of each slide was assigned values from 1~4 based on the percentage of positive cells (1, 0~24%; 2, 25~49%; 3, 50~74%; 4, 75~100%). The staining intensity was scored from 0~3 based on the color of stained slides (0, no staining signals; 1, light yellow; 2, pale brown; 3, seal brown). In this case, the IHC scores of each slide was determined by the assigned value of staining percentage multiplying that of the staining intensity. During data processing, the median of IHC scores of all slides was the cutoff value to decide the expression pattern of CDC42EP3 (≥the median IHC score, the expression pattern was high; vice versa was low) in each slide.

### Lentiviral vector

Human CDC42EP3-targeting shRNA (shCDC42EP3-1, 5′-CGGACTCTGTGTTCACAGAAA-3′; shCDC42EP3-2, 5′-AAGCTCTCATGTTGCCCTTAT-3′; shCDC42EP3-3, 5′-ATGCGAGCTCATCAAGGGAAA-3′) and three shCCND1 (shCCND1-1, 5′-GGTGAACAAGCTCAAGTGGAA-3′; shCCND1-2, 5′-CCACAGATGTGAAGTTCATTT-3′; shCCND1-3, 5′-GGTGAACAAGCTCAAGTGGAA-3′) were designed and prepared to clone into BR-V108 vectors. Age I (Cat. #R3552L, NEB) and EcoRI (Cat. #R3101L, NEB) were supplemented at both ends to complete the construction of lentiviral vector. Competent *Escherichia coli* cells (Cat. #CB104-03, TIANGEN) were transfected with shCDC42EP3/shCCND1 vector and control vector (scramble sequence, 5′-TTCTCCGAACGTGTCACGT-3′). Positive clones were then identified by PCR and plasmids were extracted using EndoFree Maxi Plasmid Kit (Cat. #DP118-2, TIANGEN). Finally, the lentivirus was packaged with prepared plasmids to construct CDC42EP3/CCND1-knockdown cell models. In addition, the sequences of CDC42EP3 or c-Myc were cloned to LV-013 plasmids (Cat. #LV-013, Shanghai Bioscienceres, China) which was further used for establishing gene overexpression cell models.

### Cell lines

Two human glioma cell lines (SHG-44 and U251) were applied to build cell models. Experimental cells in the logarithmic growth phase (2 × 10^5^) were seeded into 1640-medium containing 10% FBS (fetal bovine serum, Cat. #16000-044, Invitrogen) and 20 mL GFP (green fluorescent proteins)-lentiviral vectors (1 × 10^8^ TU/mL) at a MOI of 5 in the presence of ENI.S and Polybrene. The cells were cultured for 72 h and fresh medium containing 2 μg/ml puromycin (Cat. #A11138-003, Gibco) was added once every day. The efficiency of lentivirus infection was valued by observing GFP fluorescence under a fluorescence microscope (Cat. #IX73, OLYMPUS).

### Real-time quantitative PCR (qPCR) and chromatin immunoprecipitation assay-PCR (ChIP-PCR)

Experimental cells were collected and total RNA was collected using Trizol lysate (1 mL, Cat. #T9424-100m, Sigma). The concentration and quality of RNA were determined with Nanodrop 2000/2000c spectrophotometer. Then the reverse transcription primer (2 μL, 0.5 μg/μL) and total RNA (2.0 μg) was added into PCR tubules for reaction to obtain cDNA with Hiscript QRT supermix for qPCR (+gDNA WIPER) (Cat. #R123-01, Vazyme). Thus, prepared cDNA was used for the following PCR using AceQ qPCR SYBR Green master mix (Cat. #Q111-02, Vazyme). Lastly, 2^−ΔΔCT^ method was employed to calculate the relative mRNA expression level in each experimental cell group. GAPDH was considered as the negative control, and the upstream and downstream primer sequences were showed in Table S1. For ChIP-PCR, the ChIP-DNA complex was obtained through anti-CCND1 antibody.

### Western blotting-based assay (WB)

Experimental cells were prepared and total protein was extracted using PBS (phosphate buffer saline) and 1×lysis buffer while the concentration and quality of total protein was detected using BCA Protein Assay Kit (Cat. #23225, HyClone-Pierce). Equal amounts of total protein were separated by 10 mL SDS-PAGE (polyacrylamide gel) consisting of 30% PAGE, 1.5 mol/L Tris, 10% SDS, 10% APS and TEMED. Then, the separated protein was transferred to a polyvinylidene difluoride (PVDF) membrane. The membrane was subsequently blocked with TBST solution containing 5% skim milk for 1 h at room temperature. Primary antibodies of targeted proteins were incubated with the membrane for another 2 h at 25 °C. After washed by TBST solution for three times (10 min/time), the second antibody (HRP Goat Anti-Rabbit IgG, 1:3000, Cat. #A0208, Beyotime) was added to the membrane for 2 h incubation at room temperature. Color development of each membrane was then performed using ECL-PLUS/Kit (Cat. #RPN2232, Amersham). Lastly, the membranes were undergone X-ray developing using ImageJ software with GAPDH as the negative control for all bands. Antibodies applied in WB assay were listed in Table S2.

### CCK8 assay

Cell proliferation of SHG-44 and U251 cells harboring shCtrl/shCDC42EP3 lentivirus were examined employing MTT assay. After disposed by trypsin, experimental cells within logarithmic growth phase were seeded into five 96-well plates (~2000 cells/well) (Cat. #3599, Corning). Before the detection, 10 μL CCK8 reagent were added into each well and cultured for 4 h in an incubator until the visual color conversion occurred. Absorbance at 450 nm were measured using a microplate reader/a spectrophotometer. Viability percentage (%) = (Absorption value of supernatant of treatment group) / (Absorption value of supernatant of control group) × 100%.

### Celigo cell counting assay

In the rescue experiments, we examined cell viability using Celigo cell counting assay. The cells within logarithmic growth phase were digested with trypsin. Then cell suspension (100 µL/well) were supplemented into each well of the 96-well plates which was subsequently cultured at 37 °C with 5% CO_2_. The plates were scanned using Celigo Image Cytometer (Nexcelom) for successive five days. The curve of the change multiple of the number of cells in the experimental group and the control group with time was drawn.

### Cell colony formation

Cell colony formation of the experimental cells in the rescue experiments were also performed. Specifically, cells within the logarithmic phase were dealt with trypsin and then resuspended, counted and seeded in 6-well plates (400~1000 cells/well). After incubated for 14 days, cells were washed three times using PBS, fixed with paraformaldehyde (1 mL, 4%) for 30~60 min and then stained with Giemsa (500 µL) for 10~20 min. Subsequently, the experimental cells were disposed using ddH_2_O for several times, aired and photographed. Lastly, the amount of cell colonies was computed under a microscopy (Cat. #IX71, Olympus).

### Flow cytometry (FACS)

The influences of lentivirus transfection on cell cycle and apoptosis of experimental cells were determined using Flow cytometry. For detecting cell cycle, the cells were cultured for five days by seeding in 6 cm-well dishes. Then the cells were disposed with trypsin, washed by pre-cooled PBS (pH = 7.2~7.4) and then fixed using ethanol (70%) for 1 h. After centrifuged and washed again, cells were stained using mixed staining solution containing 40 × PI (2 mg/mL) (Cat. #P4170, Sigma), 100 × RNase (10 mg/mL) (Cat. #2158-1, TakaRa) and 1 × PBS (25:10:1000). A FACSCalibur (Guava easyCyte HT, Millipore) was lastly used to detect the number of cells in three cell cycle phases. To examine the quantity of apoptotic cells, cells were disposed with trypsin, centrifuged and washed with pre-cooled D-Hanks (pH = 7.2~7.4). Following resuspended with 1×binding buffer (200 µL), the cells were stained using Annexin V-APC single staining kit (Cat. #88-8007, eBioscience) for 10~15 min at room temperature. Then the apoptotic cells were observed under a fluorescence microscope (Cat. #IX73, Olympus) and the percentage was analyzed with the FACSCalibur.

### Wound-healing assay

The experimental cells were seeded into 96-well plates until cell confluence was up to 90%, and then the culture medium was changed to low-concentration serum medium. A 96-Wounding Replicator (Cat. #VP408FH, VP scientific) was subsequently used to scratch wounds across each cell layer and cell debris generated were washed for 2~3 times with serum-free medium. Cell layers with wounds were cultured and photos were taken 8 h/24 h after scratching under a fluorescence microscope.

### Transwell assay

The cellular ability of migration was also detected using Transwell kit (Cat. #3422, Corning). In brief, the stably-transfected cells in logarithmic growth phase were dealt with trypsin and resuspended using low-concentration serum medium. After adding 100 μL (per well) serum-free medium into a 24-well plate and culturing for 1~2 h, the medium was removed and then the lower chamber was supplemented using culture medium containing 30% FBS (600 μL). Subsequently, cell suspensions (100 μL) containing 1~2 × 10^5^ cells were added into each chamber. After culturing for 24 h or 48 h (in the rescue experiments), migratory cells adhering to the bottom of polycarbonate membrane were stained and photographed with the fluorescence microscope.

### GeneChip primeview

Six RNA samples from stably-transfected SHG-44 cells (shCDC42EP3 and shCtrl groups) were collected with Trizol reagent (Thermo Fisher Scientific, USA). The purified RNA (≥50 ng/μL) was then quantified by NanoDrop 2000 spectrophotometer (Thermo Fisher Scientific, USA) (1.7 < A260/A280 < 2.2). The gene chip analysis was undergone with TruSeq Stranded mRNA LT Sample Prep Kit (Illumina, USA) and then scanned by Affymetrix Scanner 3000 (Affymetrix, USA). The differentially expressed genes (DEGs) between shCtrl- and shCDC42EP3-harboring cells were determined (|Fold change |≥1.3 and FDR < 0.05). Ingenuity pathway analysis (IPA) (Qiagen, Germany) was performed based on all DEGs for analyzing the enriched functional annotations. The absolute value of the *Z* score greater than 2 is considered meaningful. The remaining data were analyzed for significant difference analysis and functional analysis of differential genes, including canonical pathway analysis and network analysis.

### Dual-luciferase reporter assay

Wild-type (WT) or mutated (MUT) CCND1 promotors were cloned into firefly luciferase reporter vector. Together with renilla luciferase control plasmid, it was co-transfected into SHG-44 and U251 cells with or without CDC42EP3 or c-Myc overexpression. 36 h after the transfection, cells were harvested and subjected to the measurement of luciferase activity by using a Dual-Luciferase Reporter Assay System according to the manufacturer’s manual.

### Mice xenograft experiments

To examine the effects of CDC42EP3 knockdown in vivo, we subcutaneously injected 1 × 10^7^ shCDC42EP3 or shCtrl U251 cells into 20 BALB/C nude mice (female, 4 weeks old) which were ordered from Beijing Vital River Laboratory Animal Technology Co., Ltd. Seven days after injection, we documented mouse weight, tumor length and diameter once per week. After four weeks, the animals were anesthetized following intraperitoneal injection (0.7%, 10 µL/g) of pentobarbital sodium (Sigma). The mice under anesthesia were placed under the in vivo optical imaging system (Caliper IVIS Lumina II, Cat. #LB983, Berthold Technologies) for fluorescence detection. Finally, the experimental animals were killed and tumors were removed for Ki-67 staining with Ki-67 antibody (1:300, Cat. #Ab16667, Abcam) and the second antibody (HRP Goat Anti-Rabbit IgG, 1:400, Cat. #Ab97080, Abcam).

### Co-immunoprecipitation (Co-IP) assay

Co-IP assay was implemented to detect the protein-protein interaction involved in CDC42EP3 in the experimental cells. Briefly, proteins were extracted from U251 cells and BCA (bicinchoninic) method was used to quantify protein concentration. Subsequently, protein lysate (1.0~1.2 mg) and antibodies were added for incubation at 4 °C overnight. Incubated protein was added into beads (20 μL) and PBS (1 mL) for incubation at 4 °C for 2 h. After centrifuging and washing for three times, the protein samples were incubated in IP lysate and 5×loading buffer at 100 °C for five min. Finally, proteins were separated by SDS-PAGE and quantified using WB assay described above. Antibodies applied in Co-IP assay were listed in Table S2.

### Statistical analysis and ethical approval

All cell experiments were carried out in triplicate and the data were displayed as means ± standard deviations (M ± SD). We employed Chi-square to detect the difference in expression pattern of CDC42EP3 between clinical samples and normal tissue samples. Spearman correlation test was applied to examine the correlation between CDC42EP3 expression pattern and features of clinical samples. The influence of CDC42EP3 expression pattern on the overall survival of the patients was determined using Kaplan–Meier analysis and log-rank test. Differences in cytological characteristics between shCtrl and shCDC42EP3 groups were analyzed using Student’s *t* test. All statistics and plotting were executed using SPSS 19.0 and GraphPad Prism 8.0. *P* < 0.05 was considered as statistically significant. Ethical approval of the experiments involved in this study has been acquired from Ethics committee of Shanghai Fudan University.

## Results

### CDC42EP3 is upregulated in glioma and correlated with poor prognosis

**Fig. 1:**
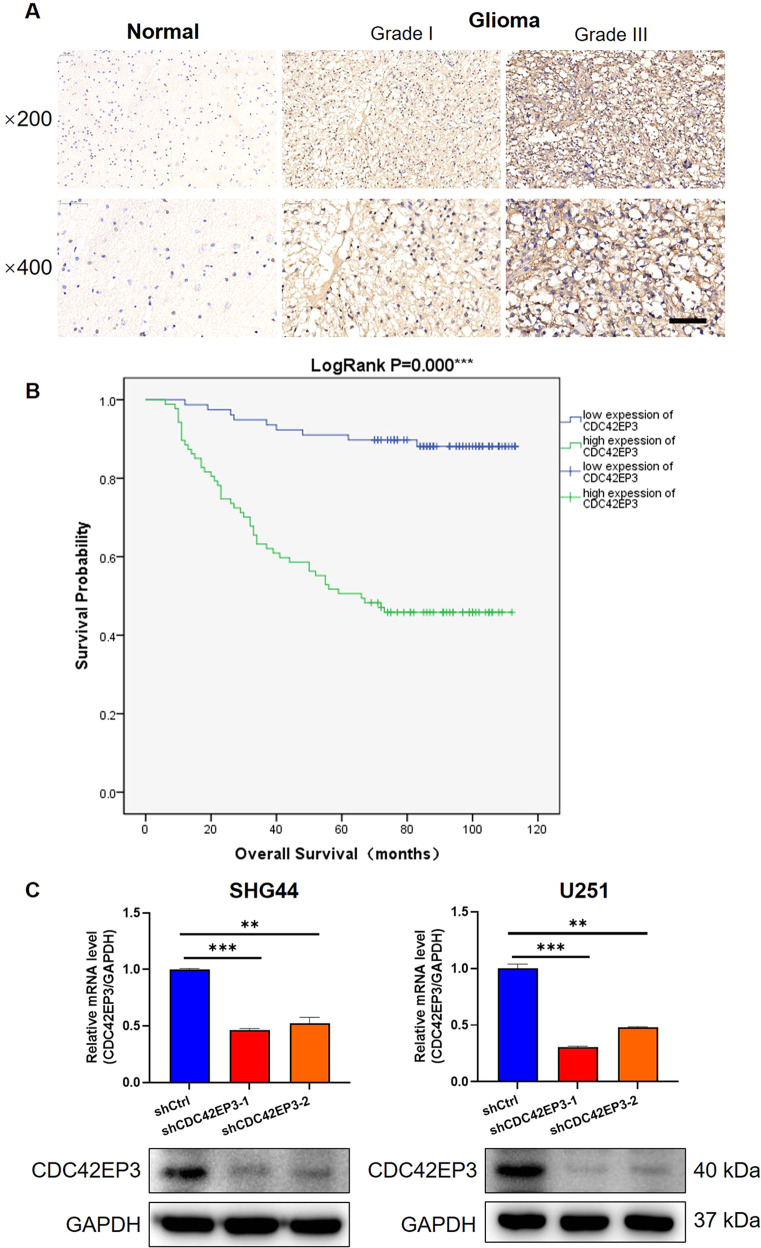
CDC42EP3 is upregulated in glioma and correlated with poor prognosis. **A** Representative photographs of human glioma tissues and normal brain tissues after immunohistochemistry staining of CDC42EP3. The scale bar is 50 µm long. **B** Glioma patients with high expression of CDC42EP3 undergoes relatively shorter overall survival in comparison with those with low CDC42EP3 expression. **C** The relative mRNA levels of CDC42EP3 in SHG-44 and U251 cells and differences between shCtrl- and shCDC42EP3-harboring cells detected by qPCR and WB. Data were presented by mean with SD (*n* ≥ 3). **P < 0.01, ***P < 0.001.

To examine the role of CDC42EP3 played in glioma cells, we firstly identified the expression level of CDC42EP3 through immunohistochemical staining on clinical samples and found that CDC42EP3 was lower expressed in normal brain tissues than that in glioma tissues (Fig. 1A). The results of statistical analysis also indicated that the expression pattern of CDC42EP3 was more likely to be high in glioma tissues than that in normal brain tissues (Table 1). Glioma patients with high expression of CDC42EP3 underwent much shorter overall survival (Fig. 1B) and more likely to suffer from tumor recurrence (*p* < 0.001, Table 2) than those with low level of CDC42EP3. In addition, the higher pathological grade of glioma was, the more likely CDC42EP3 was upregulated in patients (*p* < 0.001, Table 2). These findings were also verified by the following Spearman correlation test that the pathological grade of glioma was significantly correlated with the CDC42EP3 expression pattern (*r*_*s*_ = 0.631, *p* < 0.001, *n* = 165) and patients with high expression pattern of CDC42EP3 were more likely to recur (*r*_*s*_ = 0.513, *p* < 0.001, *n* = 165). Moreover, the analysis using RNA-seq data of TCGA also indicated the upregulation of CDC42EP3 in glioma and its correlation with poor prognosis, with an AUC (area under the ROC curves) of 0.735 (Fig. S1A–S1C). These results indicated potential involvement of CDC42EP3 in glioma progression. We thus performed further experiments to identify the specific role CDC42EP3 played in vitro and in vivo.

**Table 1:** Expression patterns of CDC42EP3 in glioma and normal tissues derived from immunohistochemistry analysis.

CDC42EP3 expression pattern	Glioma tissue		Normal tissue		*p* value
	No. of cases	Proportion	No. of cases	Proportion	
Low	78	47.3%	24	100%	<0.001
High	87	52.7%	0	0%

**Table 2:** Comparison between the expression pattern of CDC42EP3 and patients’ or tumor characteristics.

Characteristics	No. of patients	CDC42EP3 expression pattern		*p* value
		Low	High	
All patients	165	78	87	
Age (years)				0.258
≤41	86	44	42	
>41	78	33	45	
Sex				0.298
Male	100	44	56	
Female	65	34	31	
Tumor recurrence				<0.001
No	74	56	18	
Yes	91	22	69	
Grade				<0.001
I	20	18	2	
II	70	51	19	
III	51	7	44	
IV	24	2	22	
Expression of GFAP				0.151
−	2	1	1	
+	64	25	39	
++	65	31	34	
+++	11	7	4	

Note, 41 years old is the median age of the sampled patients.

*GFAP* glial fibrillary acidic protein.

### Establishment of CDC42EP3 knockdown cell models

Before in vitro experiments, we firstly built CDC42EP3-knockdown glioma cell models based on SHG-44 and U251 cells. Following the transfection of shCDC42EP3-harboring lentivirus, it was demonstrated that all 3 shRNAs present strong ability in knocking down the expression of CDC42EP3 in SHG-44 cells (Fig. S2). Among them, shCDC42EP3-1 and shCDC42EP3-2 with relatively better knockdown efficiencies were used for constructing CDC42EP3 knockdown cell models and carrying out loss-of-function experiments. As shown in Fig. 1C, results of both qPCR and WB verified the downregulation of CDC42EP3 in SHG-44 and U251 cells, suggesting the successful establishment of CDC42EP3 knockdown cell models.

### Effects of CDC42EP3 depletion on cellular phenotypes in vitro

**Fig. 2:**
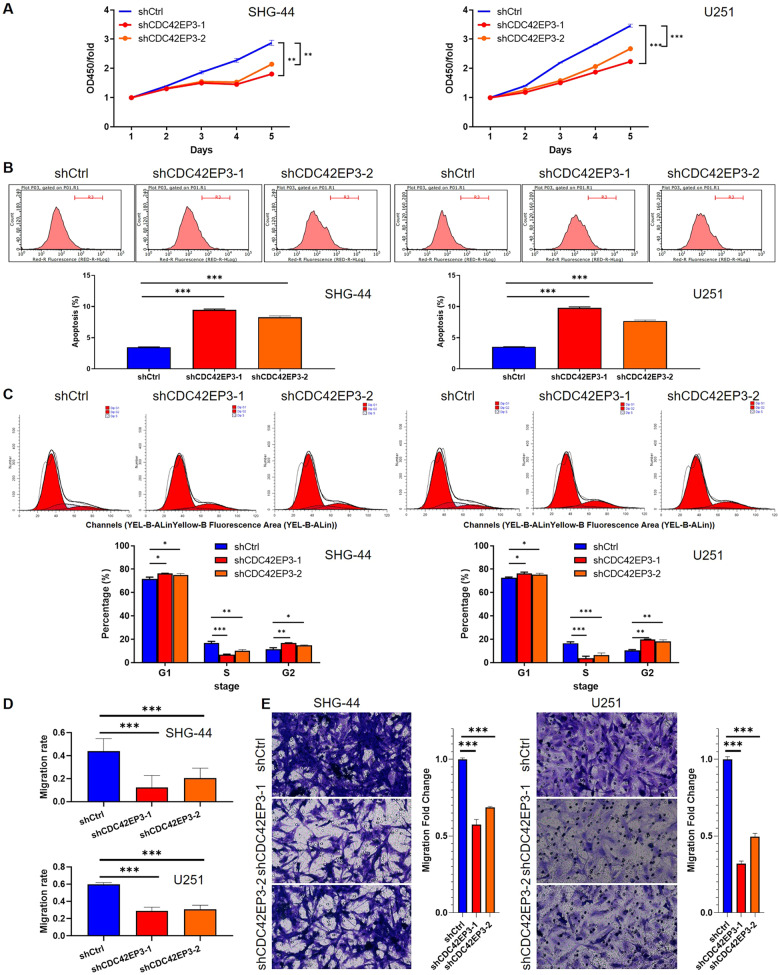
Effects of CDC42EP3 depletion on cellular phenotypes in vitro. **A** Results of CCK8 assay indicated a significant inhibition on cell proliferation after depleting CDC42EP3 in SHG-44 and U251 cells. **B** Higher level of cell apoptosis following downregulation of CDC42EP3 than controls was detected by FACS (shCtrl *vs*. shCDC42EP3) in both cell lines. **C** Comparison of percentages of cells in three cell cycle phases (shCtrl vs. shCDC42EP3) in both experimental cell lines. **D** Representative images and differences on cell migration rate detected by wound-healing assay (shCtrl vs. shCDC42EP3) in both cell lines. **E** Representative images (×200) and the comparison on cell migration ability resulted from Transwell assay on the experimental cells. The left panel lists results for SHG-44 cells and the right panel is for U251 cells. Data were presented by mean with SD (*n* ≥ 3). *P < 0.05, **P < 0.01, ***P < 0.001.

First of all, the findings of CCK8 assay displayed that both shCDC42EP3-harboring SHG-44 and U251 cells exhibited a depressed ability of proliferation in comparison with shCtrl-harboring cells (SHG-44 cells, *p* < 0.001; U251 cells, *p* < 0.001) (Fig. 2A). In addition, changes in cell apoptosis were also detected by FACS and the results manifested that downregulated CDC42EP3 mediated by lentiviral transfection significantly increased the amount of apoptosis cells compared with the negative controls (SHG-44 cells, *p* < 0.001; U251 cells, *p* < 0.001) (Fig. 2B). The percentage of cells in three cell cycle phases of shCtrl and shCDC42EP3 groups showed similar changes in both cell lines. Specifically, CDC42EP3 knockdown did not exert an influence on the quantities of G2-stage cells, but cells in S stage were significantly decreased (SHG-44 cells, *p* < 0.05; U251 cells, *p* < 0.05) with G2-stage cells increased (SHG-44 cells, *p* < 0.05; U251 cells, *p* < 0.05) in both cell lines (Fig. 2C). These results indicated that CDC42EP3 knockdown may block cell cycle in G2 stage. Moreover, cellular ability of migration identified by Wound-healing assay and Transwell assay was also remarkably altered after depletion of CDC42EP3. Specifically, shCDC42EP3-harboring cells of both cell lines showed attenuated migratory ability in comparison with shCtrl-harboring ones 48 h (SHG-44 cells, *p* < 0.001; U251 cells, *p* < 0.001) after producing the wound (Figs. S3 and 2D). Similarly, results of Transwell assay revealed a sharp decrease of cell migration in both cells (*p* < 0.001) (Fig. 2E). The above findings suggested that CDC42EP3 knockdown limited cell proliferation, cell migration but accelerated cell apoptosis in vitro.

### CDC42EP3 depletion restricted in vivo tumor growth

**Fig. 3:**
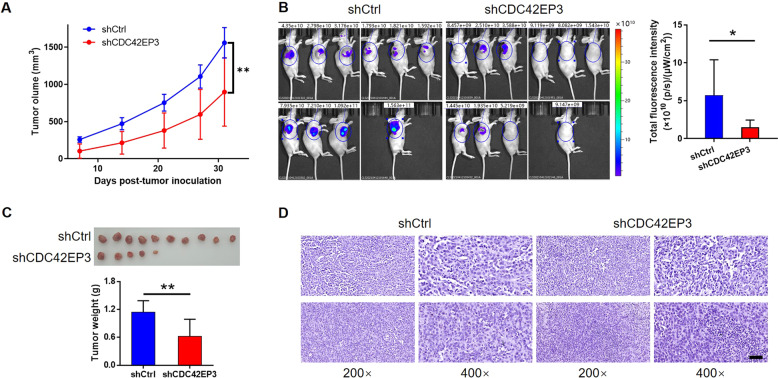
CDC42EP3 depletion restricted in vivo tumor growth. **A** The comparison on the tumor volume after injecting shCtrl- and shCDC42EP3-harboring U251 cells into experimental animals. **B** The fluorescence imaging and the difference on the total fluorescence intensity of two experimental groups (shCtrl vs. shCDC42EP3) of mice. **C** The removed xenograft tumors and the difference in tumor weights between two groups (shCtrl vs. shCDC42EP3). **D** Representative images on the expression of Ki-67 derived from Ki-67 staining in the solid tumors. The scale bar is 50 µm long. Data were presented by mean with SD (*n* ≥ 3). *P < 0.05, **P < 0.01.

To examine the potential influence of CDC42EP3 depletion on in vivo tumor growth, we established mouse models through subcutaneously injecting experimental U251 cells and recorded the growth of xenograft tumors in detail. As a result, we found that xenograft tumors in the shCDC42EP3 group grew much slower than those in the shCtrl group (Fig. 3A). In vivo fluorescence imaging on tumors also revealed that the amount of total fluorescence in the shCtrl mice was much more than those in the shCDC42EP3 group (*p* < 0.05) (Fig. 3B). After sacrificing the mice, xenografts were collected, photographed and weighed. It was unsurprising that dissociative tumors from shCDC42EP3-harboring mice were lighter than those from shCtrl-harboring animals (*p* < 0.01) (Fig. 3C). The following Ki-67 staining also verified that downregulation of CDC42EP3 restricted the growth activity of xenografts (Fig. 3D). These findings demonstrated the lowered expression of CDC42EP3 could significantly restrain glioma tumor growth in vivo.

### Exploring downstream target molecules of CDC42EP3 in glioma cells

**Fig. 4:**
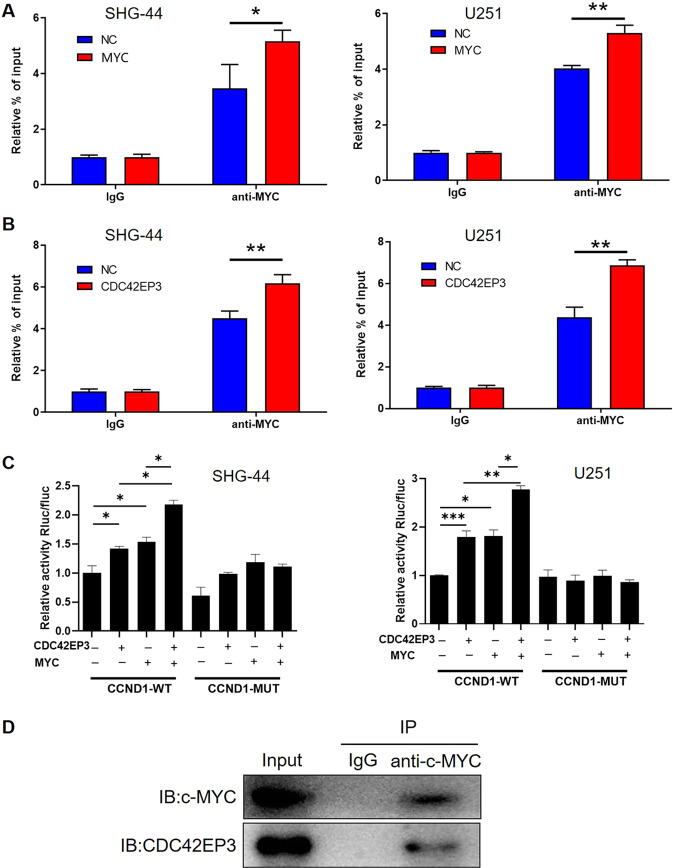
Exploring downstream target molecules of CDC42EP3 in glioma cells. **A** The volcano plot of differentially expressed genes (DEGs) profiling between shCtrl- and shCDC42EP3-harboring SHG-44 cells. Red/green dots represents DEGs with the threshold of the absolute fold change >1.3 and the FDR <0.05. **B** CDC42EP3-related interaction network and signaling pathways identified by IPA in SHG-44 cells. **C** The changes in the mRNA expression of 20 DEGs following CDC42EP3 depletion in SHG-44 cells were identified by qPCR. All relative mRNA levels of the downstream genes of CDC42EP3 were significantly different between two groups (shCtrl vs. shCDC42EP3). **D** The changes in the expression of several downstream targets following CDC42EP3 depletion in SHG-44 cells identified by WB. **E** The differential expression of CCND1 in glioma tissues and normal brain tissues was analyzed based on the data collected from TCGA database. **F** ROC curve analyses and AUC values for CCND1 in glioma and normal brain tissues. Data were presented by mean with SD (*n* ≥ 3). **P < 0.01.

To further interpret the downstream regulatory mechanism underlying the inhibitory effects of CDC42EP3 depletion on glioma, we next executed a transcriptional profiling via a gene chip using shCtrl- (*n* = 3) or shCDC42EP3-harboring (*n* = 3) SHG-44 cells. Overall, there were 7961 differentially expressed genes (DEGs) identified with 3947 DEGs upregulated and the others downregulated (Fig. 4A). The following ingenuity pathway analysis (IPA) on canonical signaling pathway showed that PI3K/AKT signaling, mTOR signaling, Wnt/β-catenin signaling and NF-κB signaling pathways were associated with CDC42EP3 depletion (Fig. 4B). We thus performed qPCR to detect the expression levels of several downstream genes of CDC42EP3 identified by IPA and uncovered that BTRC, PPP2CA, PPP2R1B, CCND1, CD44, CDC42, TRAF6, UBC, CSNK1D, HSP90AA1, HSP90AB1, JUN, KRAS and MALT1 were significantly downregulated following the knockdown of CDC42EP3 (Fig. 4C). WB assay was then carried out to determine some of the downstream protein targets and the results manifested that CCND1 were most downregulated while the level of CD44 remained unchanged in shCDC42EP3-harboring SHG-44 cells compared with negative controls (Fig. 4D). Moreover, data analysis of TCGA indicated that CCND1 also showed upregulated expression in glioma in comparison with normal brain tissues with an AUC of 0.909 (Fig. 4E, F). Collectively, the above exploration suggested that CCND1 may be a downstream effector of CDC42EP3 in glioma, which deserve further investigation. Moreover, due to the involvement of NF-κB signaling pathway in CDC42EP3-mediated regulation, we further examined the level of several inflammation-related or stemness-related proteins in U251 cells and discovered that along with CDC42EP3 knockdown, CD133, MELK, SOX2, MIP-1α were downregulated and TLR4 was upregulated with HMGB1 remaining unchanged (Fig. S4).

### CDC42EP3 regulates CCND1 expression through c-Myc-mediated transcription

**Fig. 5:**
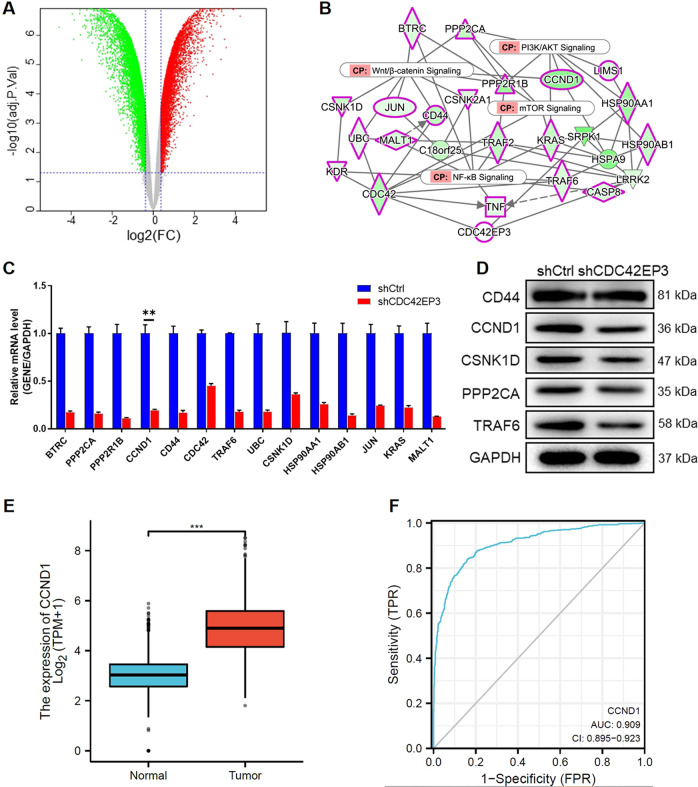
CDC42EP3 regulates CCND1 expression through c-Myc-mediated transcription. **A** ChIP assay confirmed the binding of c-Myc with CCND1 promotor, which could be further enhanced by c-Myc overexpression. **B** Results of ChIP assay showed that the binding of c-Myc with CCND1 promotor could be enhanced by CDC42EP3 overexpression. **C** Dual-luciferase reporter assay indicated the interaction between c-Myc and CCND1 promotor (wild type or mutated) in SHG-44 and U251 cells with or without c-Myc or CDC42EP3 overexpression. **D** A co-immunoprecipitation assay was performed to verify the protein–protein interaction between CDC42EP3 and c-Myc. Data were presented by mean with SD (*n* ≥ 3). *P < 0.05, **P < 0.01, ***P < 0.001.

In order to explore the molecular mechanism by which CDC42EP3 regulates CCND1, EPD The Eukaryotic Promotor Database (https://epd.epfl.ch//index.php) and Animal Transcription Factor Database (http://bioinfo.life.hust.edu.cn/AnimalTFDB#!/) were used for obtaining CCND1 promotor and predicting transcription factor of CCND1, respectively. Correspondingly, oncogene c-Myc was identified as a candidate, the binding of which with CCND1 promotor was confirmed by ChIP in both SHG-44 and U251 cells (Fig. 5A). As a well-known oncogene, TCGA data showed that high expression of c-Myc was also found to be associated with poor prognosis of glioma patients with an AUC of 0.992 (Fig. S5A, B), which was also positively correlated with CCND1 expression (Fig. S5C). More importantly, CDC42EP3 overexpression could significantly enhance the interaction between c-Myc and CCND1 promotor (Fig. 5B). Based on the binding site motif on CCND1 with c-Myc predicted by JASPAR database (Fig. S6), a promotor-mutant CCND1 (CCND1-MUT) was constructed together with wild type (CCND1-WT) for luciferase reporter gene assay. As shown in Fig. 5C, overexpression of c-Myc or CDC42EP3 could both facilitate the interaction between c-Myc and promotor of CCND1-WT but not CCND1-MUT. All above results suggested that CDC42EP3 may regulate CCND1 expression through c-Myc-mediated transcription. Furthermore, a co-IP assay revealed the protein–protein interaction between CDC42EP3 and c-Myc, providing molecular basis for the regulation of c-Myc-mediated transcription of CCND1 by CDC42EP3 (Fig. 5D).

### CCND1 depletion mitigates pro-tumor effects induced by CDC42EP3 overexpression

**Fig. 6:**
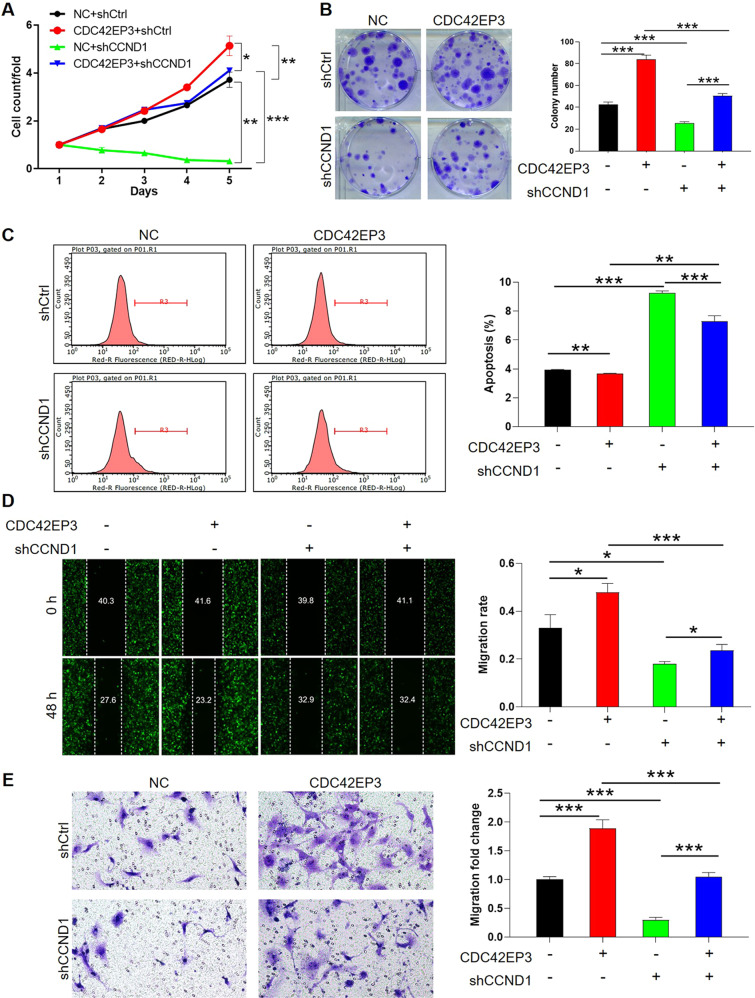
CCND1 depletion mitigates pro-tumor effects induced by CDC42EP3 overexpression. CDC42EP3 overexpression (CDC42EP3 group), CCND1 knockdown (shCCND1 group) and CDC42EP3 overexpression + CCND1 knockdown (CDC42EP3 + shCCND1 group) cell models were constructed to carry out functional rescue experiments. **A** The comparison of cell proliferation between four cell groups resulted from Celigo cell counting assay. **B** Cell colony formation and the difference in colony numbers of four cell groups. **C** The comparison between percentage of apoptotic cells in four cell groups, derived from FACS. **D** Cellular ability of migration identified by wound-healing assay and the comparison of migration rate between different cell groups. **E** Representative images in Transwell assay and the differences in migration rate between four experimental cell groups. *P < 0.05, **P < 0.01, ***P < 0.001.

Rescue experiments were finally executed to verify the role played by CDC42EP3/CCND1 axis in human glioma U251 cells. After the verification of CCND1 knockdown by 3 shRNAs targeting CCND1 silencing and the cell proliferation inhibition by shCCND1-1 and shCCND1-3 to avoid off-target effects, shCCND1-1 with relatively better efficiency was selected for the following experiments (Fig. S7A, B). After constructing CDC42EP3 overexpression (CDC42EP3), CCND1 knockdown (shCCND1) and simultaneous CDC42EP3 overexpression and CCND1 knockdown (CDC42EP3 + shCCND1) U251 cell models, we firstly determined the protein levels through WB (Fig. S7C). Next, through celigo cell counting assay, the findings indicated cell proliferation was facilitated by overexpressed CDC42EP3 and inhibited by silenced CCND1, while silencing of CCND1 relieved promotion of tumor cell growth caused by CDC42EP3 overexpression (Figs. S7D and 6A). In addition, although lowered expression of CCND1 imposed significant restrictions on cell colony formation (*p* < 0.001), upregulating CDC42EP3 could considerably reverse this inhibitory effect (*p* < 0.001) (Fig. 6B). Moreover, cell apoptotic status of experimental cells was examined by FACS and we found that CDC42EP3 overexpression brought down the percentage of apoptotic cells in the condition of CCND1 depletion (*p* < 0.001) (Fig. 6C). Regarding cellular ability of migration, the outcomes of wound-healing assay also revealed promoting effects by CDC42EP3 overexpression (*p* < 0.05) and inhibitory effects by CCND1 knockdown (*p* < 0.05), while overexpressed CDC42EP3 increased the migratory capacity of CCND1-knockdown U251 cells (*p* < 0.05) (Fig. 5D). Transwell assay exhibited similar results to wound-healing assay (Fig. 5E). On the other hand, considering the significant regulation of cell phenotypes by CDC42EP3 knockdown, it was demonstrated that the regulatory effects of CDC42EP3 knockdown on glioma failed on condition of CCND1 overexpression, indicative of the key role played by CCND1 in downstream of CDC42EP3 (Fig. S8–S10). All of the results above indicated that knocking down CCND1 retarded pro-tumor effects mediated by upregulated CDC42EP3 in glioma. In view of the positive correlation between c-Myc and CCND1, it was demonstrated that tumor-inhibiting effects of CDC42EP3 knockdown also lose efficacy upon overexpressing c-Myc (Fig. S11).

## Discussion

In this study, we found a much higher possibility of CDC42EP3 upregulation in glioma tissues than that in normal brain tissues. In addition, the higher grade of glioma was, the more likely CDC42EP3 was overexpressed in the clinical samples. Glioma was more likely to recur when the level of CDC42EP3 was elevated. After infecting CDC42EP3-targeting lentivirus into human glioma SHG-44 and U251 cells, we established CDC42EP3-depleting cell models and found that lowered CDC42EP3 confined cell proliferation and migration while amplified cell apoptosis via trapping cell cycle in G2 stage. The inhibition in tumor growth led by CDC42EP3 depletion was also proved in xenograft mouse models. Moreover, the detection in downstream mechanism displayed that several canonical signaling pathways (i.e., Wnt/β-catenin, mTOR, PI3K/AKT and NF-κB cascades) were involved in the regulation of CDC42EP3 in SHG-44 cells. Specifically, CDC42EP3 may perform a function through regulating c-Myc-mediated transcription of CCND1 in both experimental cell lines and tumor-promoting effects were significantly suppressed following knockdown of CCND1 in U251 cells. As a result, our findings indicated that CDC42EP3 may be a tumor-promoting factor in glioma progression via mediating CCND1.

A previous study has suggested that CDC42EP3 was identified as a regulator of cancer-associated fibroblast (CAF) functions [13]. As one of the brain-specific cytoskeletal proteins, GFAP (glial fibrillary acidic protein) plays an important role in maintaining the structural and functional integrity and is regarded as an essential biomarker for neurological disorders [17]. Although previous researchers have demonstrated that GFAP in patients with higher-grade gliomas was more expressed than those with lower-grade gliomas [18], we found a different result that the protein level of GFAP was unrelated with the possibility of CDC42EP3 overexpression with the latter positively correlating with tumor pathological grading. The lack of relations between the possibility of CDC42EP3 upregulating and GFAP expression indicated that tumor-promoting effects mediated by CDC42EP3 overexpression may be independent of its regulation on coordinating actin and septin cytoskeleton rearrangements in glioma CAFs.

CCND1 (cyclin D1) is one of the regulators of CDK kinases by forming complexes with CDK4 or CDK6 as a subunit [19], whose tumor-promoting role in the development of human cancers has been intensively investigated [20]. On one hand, CCND1-dependent transcriptional program has been found to play an important role in the oncogenesis and tumor development and even could predict clinical outcome of some types of cancer such as mantle cell lymphoma [21, 22]. On the other hand, the expression of CCND1 was also found to be commonly regulated through transcription-related pathway. For example, a homeobox transcription factor NKX2-1 was found to be involved in both the development and metastasis of lung adenocarcinoma and the activation of CCND1 through interacting with its promotor [23]. HER2/HER3 were also found to be upstream transcription factor of CCND1 in lung cancer which is reported to enhance EGFR-TKI resistance [24]. In clear cell renal cell carcinoma, transcription factor NFYA was reported to be able to simultaneous transactivating CCND1 and CDK4 to promote G1/S cycle transition thus accelerating tumor development and predicting poor prognosis [25]. Moreover, a zinc finger transcription factor, EGR1, was found to be conducive to cell proliferation through specifically binding with CCND1 promoter and raising CCND1 expression in U251 cells [26]. Herein, we predicted through bioinformatics analysis and confirmed through ChIP that c-Myc could transactivate CCND1 expression, which could be enhanced by the overexpression of CDC42EP3. Also, we found downregulating CCND1 mitigated pro-tumor effects mediated by CDC42EP3 overexpression. Additionally, it was demonstrated that overexpression of CCND1 or c-Myc could invalidate the tumor-inhibiting effects of CDC42EP3 knockdown. All the results provide solid evidence that the regulation of glioma development by CDC42EP3 depends on CCND1 which is transcriptional activated by c-Myc.

In mammalian cells, proteins involved in NF-κB signaling pathway are a group of transcription factors regulating cellular immune and inflammatory properties [27]. It has also been suggested that proteins regulating cell inflammation are actively expressed in glioma cell lines which play synergistic roles in cell growth, invasion and angiogenesis in in vitro experiments [28]. In our study, we also identified the alterations in NF-κB signaling pathway in the SHG-44 shCDC42EP3 cell group. Inflammation-associated proteins, TLR4, MIP-1α and HMGB1 were dysregulated in U251 cells accompanied by transfection of shCDC42EP3-harboring lentivirus. It was suggested that regulation of CDC42EP3 in human glioma cells had something to do with the canonical NF-κB inflammatory pathway. Besides, our findings also uncovered that regulation of CDC42EP3 may be associated with stemness of human glioma cells. Specifically speaking, the stemness-related regulators, CD133, MELK and SOX2 were substantially downregulated following transfection of shCDC42EP3-harboring lentivirus into SHG-44 cells, indicating concurrent reduction in expression of CDC42EP3 and stemness of glioma stem cells. It is generally well-known that SOX2 is a critical tumor stem cell marker and maintains stem cell like phenotype [29]. All the findings above gave a hint that the high level of CDC42EP3 may be conducive to elevated self-renewal potential and tumorigenicity of glioma stem cells [30]. However, more detailed exploration about the effects of CDC42EP3 expression on inflammatory responses and stemness of stem cells in glioma cells are still required before coming to a conclusion.

In summary, we demonstrated that CDC42EP3 downregulation inhibited the malignant behaviors of glioma cells through regulating c-Myc-mediated transcription of CCND1. Our findings in this study gained better insights into CDC42EP3-mediated glioma development and offered substantial theoretical basis in glioma etiology. Further studies on detailed underlying regulatory pathways are required for potentially identifying CDC42EP3 as a therapeutic target in glioma treatment.

## Supplementary information

Supplement materials

aj-checklist

Original data of WB and PCR

## Data Availability

All data analysis is showed in this article.
