# Supplementary material for: CDC42EP3 promotes glioma progression via regulation of CCND1
Source: Cell Death Dis. 2022 Apr 1;13(4):290. doi: 10.1038/s41419-022-04733-9 (PMC8975815; doi:10.1038/s41419-022-04733-9)
Supplement: Supplementary file 1 — Supplement materials [file 41419_2022_4733_MOESM1_ESM.docx]

**
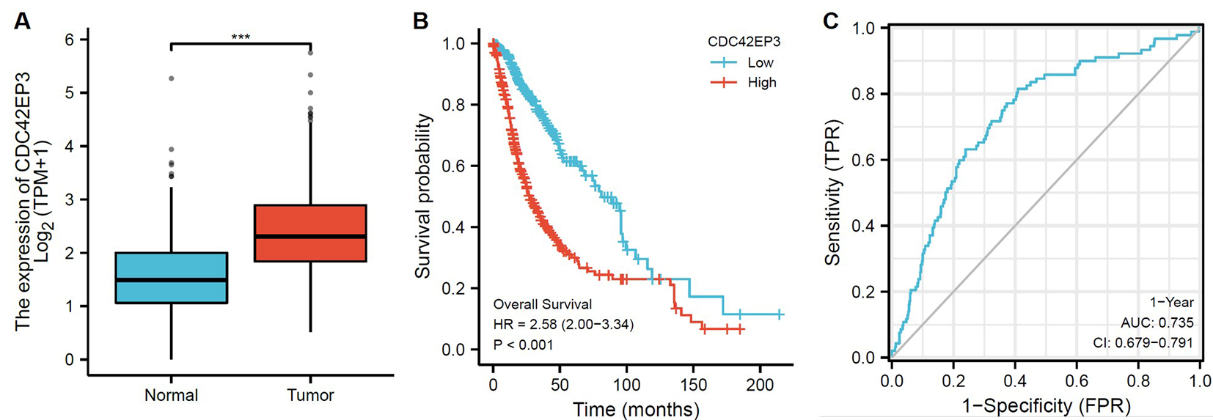
**

**Figure S1** **A.** The differential expression of CDC42EP3 in glioma tissues and normal brain tissues was analyzed based on the data collected from TCGA database. **B.** The correlation between CDC42EP3 expression and patients’ survival was analyzed based on TCGA data. **C.** ROC curve analyses and AUC values for CDC42EP3 in glioma and normal brain tissues.


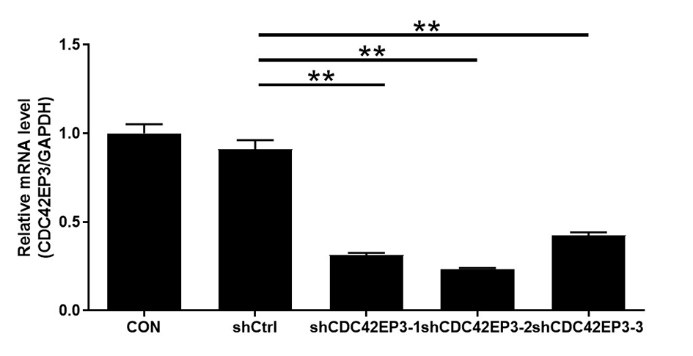


**Figure S2** The comparison on knockdown efficiencies of three shRNAs targeting CDC42EP3 in U251 cells. Data were presented by mean with SD (n ≥ 3). ***P* < 0.01.


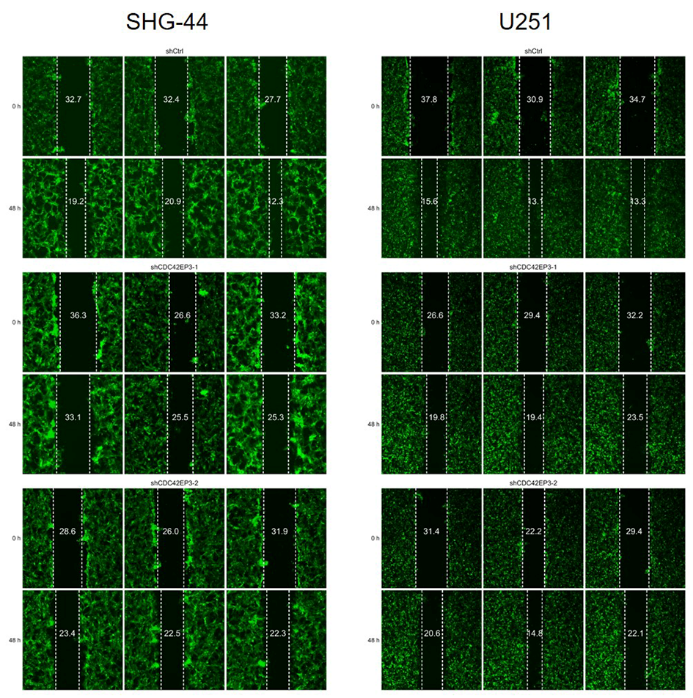


**Figure S3** Representative images on cell migration rate detected by wound-healing assay (shCtrl vs. shCDC42EP3) in SHG-44 and U251 cell lines.


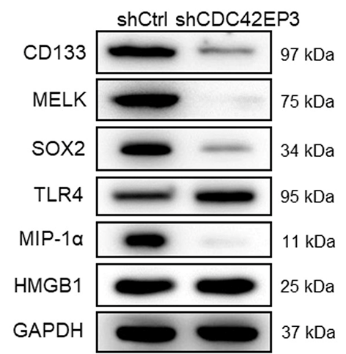


**Figure S4** The expression of inflammation-related and stemness-related proteins after knocking down CDC42EP3 in U251 cells, determined by WB.


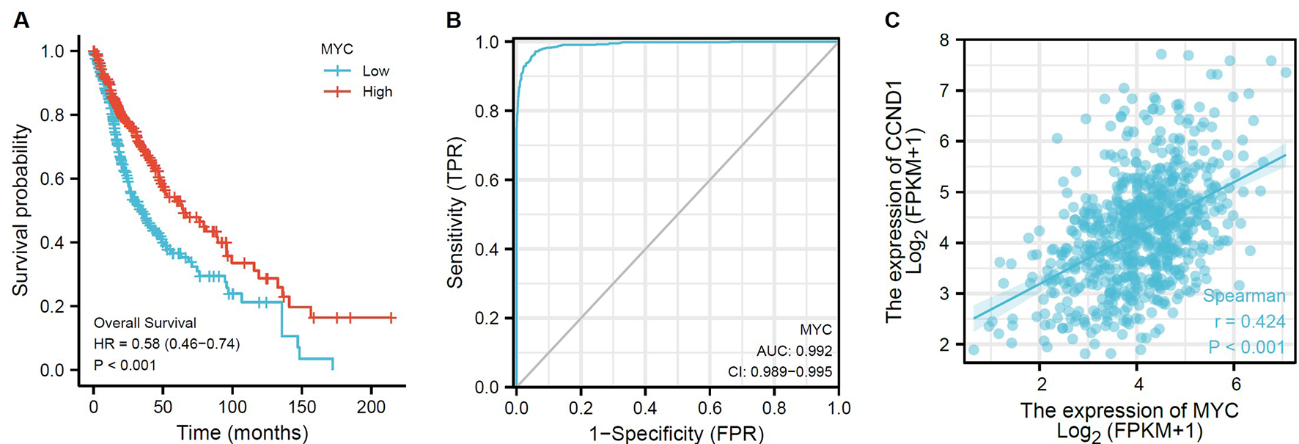


**Figure S5** **A.** The correlation between c-Myc expression and patients’ survival was analyzed based on TCGA data. **B.** ROC curve analyses and AUC values for c-Myc in glioma. **C.** The correlation between c-Myc and CCND1 was analyzed based on TCGA data.


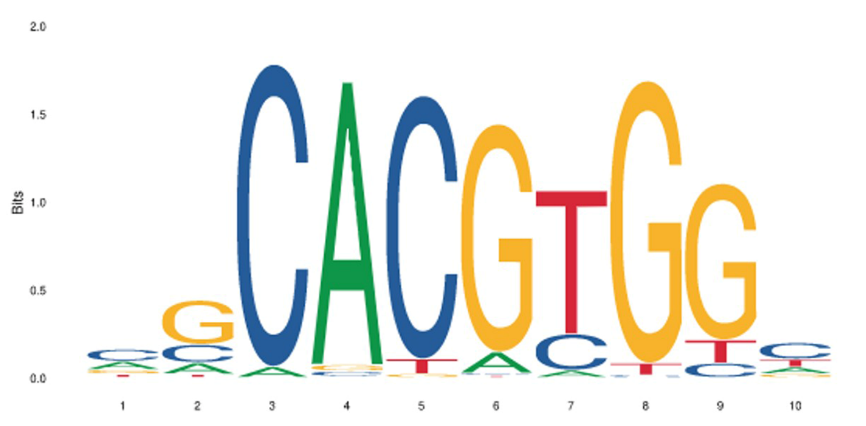


**Figure S6** The binding site motif on CCND1 promotor with c-Myc was predicated by JASPAR database.


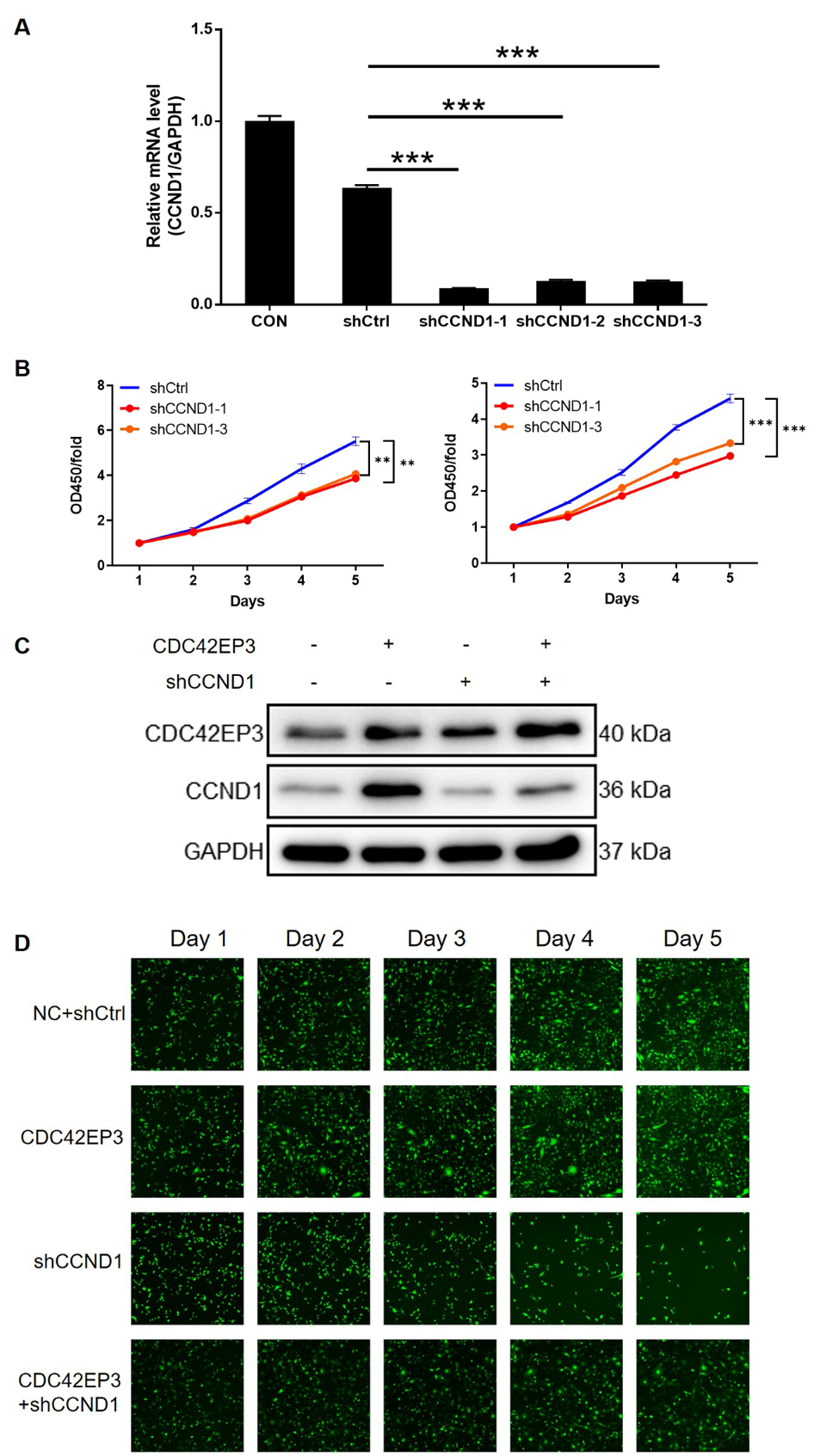


**Figure S7** **A.** The comparison on knockdown efficiencies of three shRNAs targeting CCND1 in U251 cells. **B.** The effects of shCCND1-1 and shCCND1-3 on SHG-44 and U251 cell proliferation were confirmed for avoiding off-target effect. **C.** The results of WB assay indicate the success construction of CDC42EP3 overexpression (CDC42EP3 group), CCND1 knockdown (shCCND1 group) and CDC42EP3 overexpression + CCND1 knockdown (CDC42EP3+shCCND1 group) cell models. **D.** Fluorescence images derived from celigo cell counting assay in the rescue experiments, indicating cell viability for five consecutive days in four experimental cell groups. Data were presented by mean with SD (n ≥ 3). ***P* < 0.01, ****P* < 0.001.


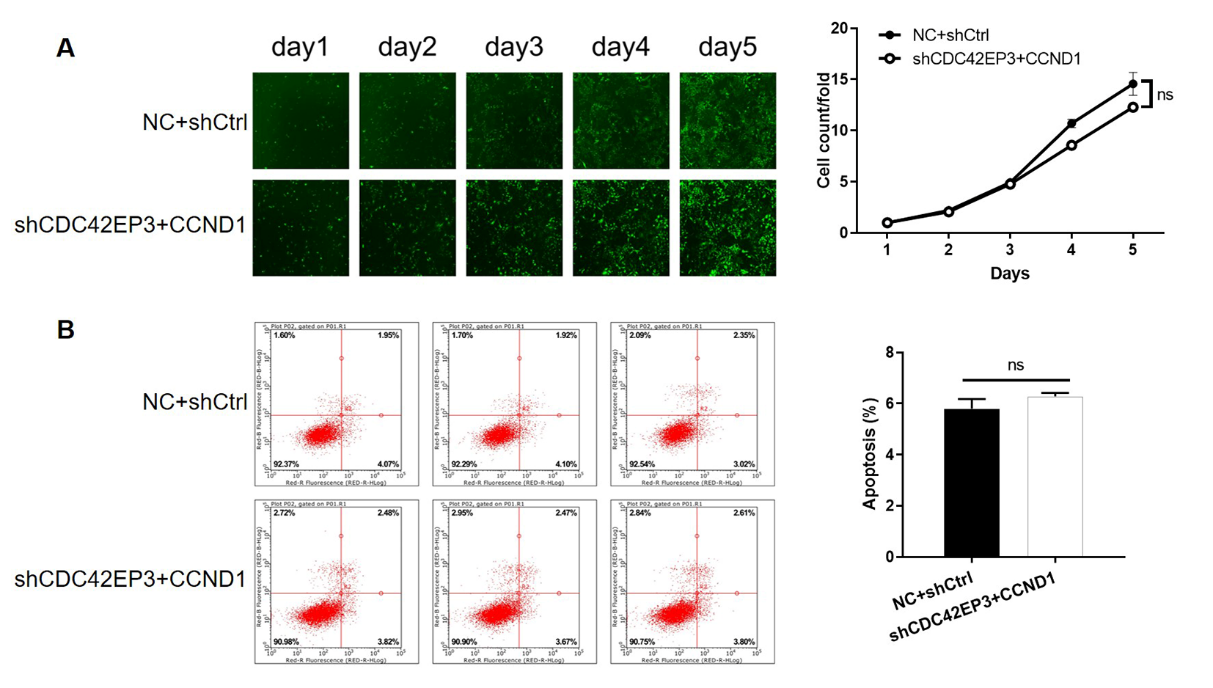


**Figure S8** CDC42EP3 knockdown + CCND1 overexpression (shCDC42EP3+CCND1 group) cell model was constructed to carry out the detection of cell proliferation (**A**) and cell apoptosis (**B**). Data were presented by mean with SD (n ≥ 3). ns = not significant.


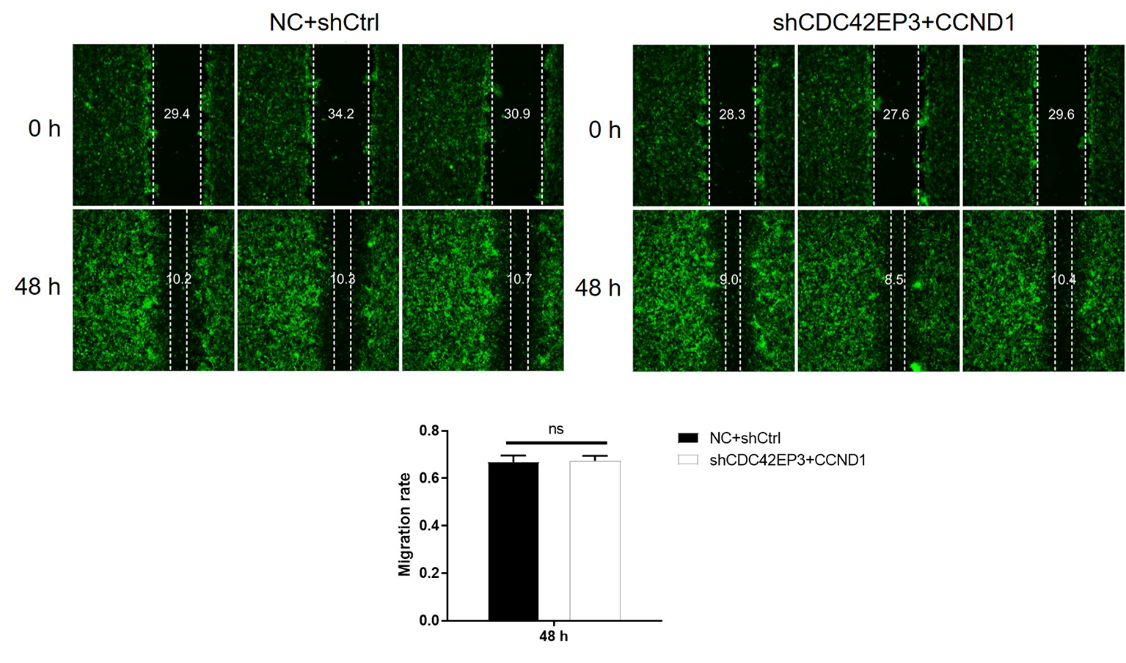


**Figure S9** CDC42EP3 knockdown + CCND1 overexpression (shCDC42EP3+CCND1 group) cell model was constructed to carry out the detection of cell migration by wound-healing assay. Data were presented by mean with SD (n ≥ 3). ns = not significant.


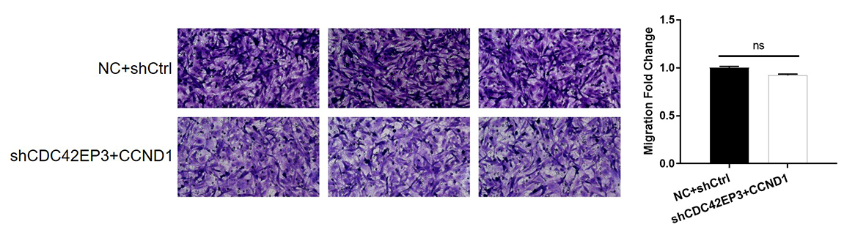


**Figure S10** CDC42EP3 knockdown + CCND1 overexpression (shCDC42EP3+CCND1 group) cell model was constructed to carry out the detection of cell migration by Transwell assay. Data were presented by mean with SD (n ≥ 3). ns = not significant.


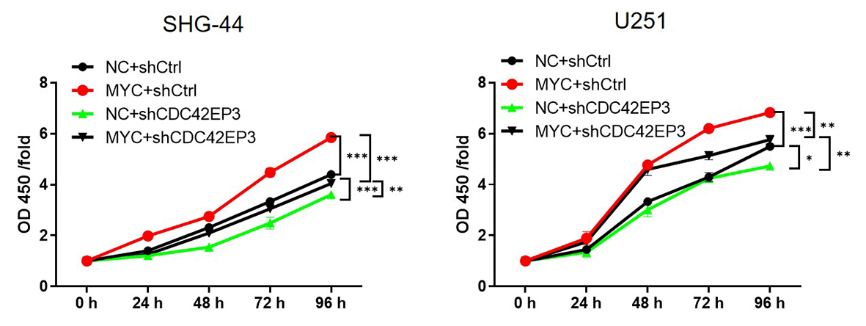


**Figure S11** CDC42EP3 knockdown (shCDC42EP3 group), c-Myc overexpression (MYC group) and CDC42EP3 knockdown + c-Myc overexpression (MYC+shCDC42EP3 group) cell models were constructed to carry out the detection of cell proliferation in both SHG-44 and U251 cells. Data were presented by mean with SD (n ≥ 3). **P* < 0.05, ***P* < 0.01, ****P* < 0.001.

**Supplementary Tables**

**Table S1** Primers used in qPCR in this study.

|  | Primer name | Upstream primer sequence (5’-3’) | Downstream primer sequence (5’-3’) |
| --- | --- | --- | --- |
| Target genes | CDC42EP3 | AGCAGTCTGTTGGAGAATGGG | AGGAGGGAACCTGTAAGGTCAG |
|  | CCND1 | AGGCGGAGGAGAACAAACAGA | GGAGGGCGGATTGGAAATGAA |
|  | GAPDH | TGACTTCAACAGCGACACCCA | CACCCTGTTGCTGTAGCCAAA |
| Downstream genes of CDC42EP3 | BTRC | TCTGTCATACCTGGATGCCAAATC | ATGCCATCAGAGGTCACTCG |
|  | PPP2CA | GTTCCCCATGAGGGTCCAAT | TTGCCCAAAGGTGTAACCAG |
|  | PPP2R1B | TTAGCAAGTGGGGATTGGTTC | GCATTTGATGCCCTGGGATAG |
|  | CCND1 | AGCTGTGCATCTACACCGAC | GAAATCGTGCGGGGTCATTG |
|  | CD44 | TGGGTTCATAGAAGGGCACG | ATACTGGGAGGTGTTGGATGTG |
|  | CDC42 | TGAAGGCTGTCAAGTATGTGG | CTCTTCTTCGGTTCTGGAGG |
|  | TRAF6 | ATTCTACACTGGCAAACCCG | CAAGGGAGGTGGCTGTCATA |
|  | UBC | CAGCCGGGATTTGGGTCG | CACGAAGATCTGCATTGTCAAGT |
|  | CSNK1D | GCTGCTTGCTGACCAAATGA | GAGGAAGTTGTCTGGCTTCACA |
|  | HSP90AA1 | TTGTAGACTGCCGAGTAATAGCC | TCCTCATCGCTGCCACTAA |
|  | HSP90AB1 | ATTGTGACCAGCACCTACGG | CATGGTGGAGTTGTCCCGAA |
|  | JUN | TGCCTCCAAGTGCCGAAAA | TAAGCTGTGCCACCTGTTCC |
|  | KRAS | GACTGGGGAGGGCTTTCTTT | CTAAGTCCTGAGCCTGTTTTGTG |
|  | MALT1 | AAGGTACTGGAGCCTGAAGGA | TCAATTCTGTGACTGTGCAACC |
|  | GAPDH | TGACTTCAACAGCGACACCCA | CACCCTGTTGCTGTAGCCAAA |

**Table S2** Antibodies involved in western blotting-based assay and co-IP assay.

| Assay | First antibody | Size/kDa | Diluted multiples | Source | Company | Serial number |
| --- | --- | --- | --- | --- | --- | --- |
| Western blot | CDC42EP3 | 40/34 | 1:1000 | Rabbit | NOVUS | NBP1-88382 |
|  | CD44 | 81 | 1:2000 | Rabbit | Abcam | Ab157107 |
|  | CCND1 | 36 | 1:2000 | Rabbit | Abcam | Ab134175 |
|  | CSNK1D | 47 | 1:1000 | Mouse | Abcam | Ab85320 |
|  | PPP2CA | 35 | 1:1000 | Rabbit | Abcam | Ab32141 |
|  | TRAF6 | 58 | 1:1000 | Rabbit | Abcam | Ab33915 |
|  | CD133 | 97 | 1:1000 | Rabbit | Abcam | Ab19898 |
|  | MELK | 75 | 1:1000 | Rabbit | Abcam | Ab108529 |
|  | SOX2 | 34 | 1:1000 | Rabbit | Abcam | Ab92494 |
|  | TLR4 | 95 | 1:1000 | Mouse | Santa Cruz | SC-293072 |
|  | MIP-1α | 11 | 1:1000 | Rabbit | Abcam | Ab25128 |
|  | HMGB1 | 25 | 1:1000 | Rabbit | Abcam | Ab18256 |
|  | GAPDH | 37 | 1:3000 | Rabbit | Bioworld | AP0063 |
| Co-IP | CDC42EP3 | 40 | 1:1000 | Rabbit | NOVUS | NBP1-88382 |
|  | CCND1 | 57-65 | 1:1000 | Rabbit | CST | 5605 |
|  | GAPDH | 36 | 1:30000 | Mouse | Proteintech | 60004-1-lg |
